# Robbing the Hospitals

**Published:** 1916-01-22

**Authors:** 


### THE EDITOR'S LETTER-BOX.

### Robbing the Hospitals.

*To the Editor of* The Hospital.

Sir,—I see that the London Homœopathic Hospital has been robbed by a clerk. I think it would be useful if, when these robberies occur, you would publish how the swindler worked his swindle. Last year the secretary of the Forget-Me-Not Bond of the London Hospital, an organisation of mine apart from the hospital, stole a very large sum. He used to give me the names of his employees, and I gave him a cheque to pay their wages; £3 a week each were thus paid for over two years to men who had no existence except in his criminal imagination. He presented bills for "work done," for which he also obtained cheques. The wage-earners' and the workdoer receipts were forged, and, of course, passed me and the auditors.

The remedy for this is never to allow the head of a department who has the engaging of the men to pay the wages. The wages should be paid by some other official—the cashier for choice. Every hospital ought to see to this at once. Experience is expensive to buy, and had better be paid for by someone else than oneself.—Yours,

61 Eaton Place, S.W. Knutsford.

[*** We should be glad to publish "how the swindler worked his swindle" on every occasion if those who know the facts will send them for publication. The difficulty heretofore has been to obtain the facts.—Ed. The Hospital.]

